# Multi-Objective Detection of River and Lake Spaces Based on YOLOv11n

**DOI:** 10.3390/s26041274

**Published:** 2026-02-15

**Authors:** Ling Liu, Tianyue Sun, Xiaoying Guo, Zhenguang Yuan

**Affiliations:** 1 College of Computer and Information Engineering, Tianjin Agricultural University, Tianjin 300392, China; liuling@tjau.edu.cn (L.L.); suntianyue0428@163.com (T.S.); 2 College of Water Conservancy Engineering, Tianjin Agricultural University, Tianjin 300392, China; 3 Tianjin Jinnan District Water Affairs Center, Tianjin 300350, China; yuanzhg@163.com

**Keywords:** four chaos, multi-target detection, YOLO v11n, deep learning

## Abstract

In response to the challenges of target recognition and misjudgment caused by varying target scales, diverse shapes, and interference such as lake surface reflections in river and lake scenarios, this paper proposes the YOLO v11n-DDH model for fast and detection of spatial targets in river and lake environments. The model builds upon YOLO v11n by introducing the Dynamic Snake Convolution (DySnakeConv) to enhance the ability to extract detailed features. It integrates the Deformable Attention Mechanism (DAttention) to strengthen key features and suppress noise, while combining the improved High-Level Screening Feature Pyramid Network (HSFPN) structure for multi-level feature fusion, thus improving the semantic representation of targets at different scales. Experiments on a self-constructed dataset show that the precision, recall, and mAP of the YOLO v11n-DDH model reached 88.4%, 78.9%, and 83.9%, respectively, with improvements of 3.4, 2.9, and 2.5 percentage points over the original model. Specifically, DySnakeConv increased mAP@50 by 0.6 percentage points, DAttention improved mAP@50 by 0.3 percentage points, and HSFPN contributed to a 0.9 percentage point rise in mAP@50. This patrol system can effectively identify and visualize various pollutants in river and lake areas, such as underwater waste, water quality pollution, illegal swimming and fishing, and the “Four Chaos” issues, providing technical support for intelligent river and lake management.

## 1. Introduction

The core challenges faced by river and lake ecosystems are primarily reflected in the issues of “four chaos” (illegal occupation, illegal extraction, illegal dumping, and illegal construction) [1] and the compound effects of multiple pollution sources [2]. Among these, the phenomena of illegal occupation, illegal extraction, illegal dumping, and illegal construction severely encroach on ecological spaces and pose systemic threats to flood prevention, navigation, and ecological security. At the same time, pollution sources are becoming increasingly complex: solid waste such as underwater accumulated plastic, construction debris, discarded fishing nets, and sunken ships release harmful substances through long-term degradation, directly harming aquatic life. In addition to traditional point-source and non-point-source pollution, there are also added risks from the decomposition products of underwater waste, illegal discharges, and disturbances from illegal activities. This results in more intense water quality fluctuations, leading to localized eutrophication, heavy metal contamination, and other risks, thus threatening drinking water safety and biodiversity. Furthermore, illegal swimming and fishing activities in non-designated areas present both safety risks and ecological damage. However, a major challenge that remains to be addressed is the difficulty in target identification and misjudgment due to interference from varying target scales, diverse shapes, and lake surface reflections in river and lake scenarios. Additionally, most existing technological approaches are developed to address specific problems within river and lake spaces, limiting their application scope.

To achieve autonomous detection within river and lake management areas, traditional methods often use color space transformation and threshold segmentation [3], morphological operations [4], and feature extraction techniques such as aspect ratio and moment features [5] to suppress lake surface reflections and lighting interference. However, their performance is limited in complex environments. In recent years, detection methods based on deep learning have gradually become mainstream, such as improved YOLO [6], R-CNN [7], and other series of algorithms. By introducing attention mechanisms [8], optimizing feature fusion networks [9], and designing specialized loss functions [10], the accuracy and robustness of multi-scale target detection have been significantly improved, and these methods have been successfully applied on embedded platforms for real-time monitoring. This study focuses on the first- and second-level rivers in Xiqing District and Binhai New Area of Tianjin, collecting images of the “four chaos” phenomena (illegal occupation, illegal extraction, illegal dumping, and illegal construction) as well as underwater plastic waste, water pollution, illegal fishing, and swimming. Images from different areas and angles of the river are obtained through on-site photography and drone aerial photography. Using the YOLO v11n-DDH algorithm, which integrates dynamic snake convolution (DySnakeConv), deformable attention mechanism (DAttention), and an improved high-level filtering feature pyramid network (HSFPN), the study aims to enhance the identification and processing efficiency of pollution issues in river and lake management areas. This provides critical technical support for achieving smart governance of river and lake spaces.

## 2. Materials and Methods

### 2.1. Data Sources

This study constructed the dataset “Space-hehu” aimed at enhancing sample diversity to improve the model’s generalization ability and robustness. The objective of constructing this dataset is to address the problem of multi-target detection and state analysis in real, open-water environments. A vision system designed for general monitoring in open waters must possess the capability to process multi-source, heterogeneous data. The dataset consists of five parts: (1). The “four chaos” dataset, which includes images captured during exploration and inspection of first- and second-level rivers in Xiqing District, Tianjin, as well as the publicly available FloW-Img dataset of floating river waste from Oka Zhibo Company, Xi’an, China. This dataset includes waste on the riverbank, floating debris (such as bottles, packaging, branches, lotus leaves, and other trash), engineering, greenhouses, and buildings. (2). The water quality and (3). The fishing dataset was captured during river and lake inspections in Binhai New Area, Tianjin. (4). The underwater plastic waste dataset consists of the J-EDI dataset, which includes deep-sea videos and images developed by the Japan Agency for Marine-Earth Science and Technology (5). The swimming dataset was collected via drone sampling, with collection scenes including rivers, beaches, and water parks.

### 2.2. Data Preprocessing

**Figure 1:**
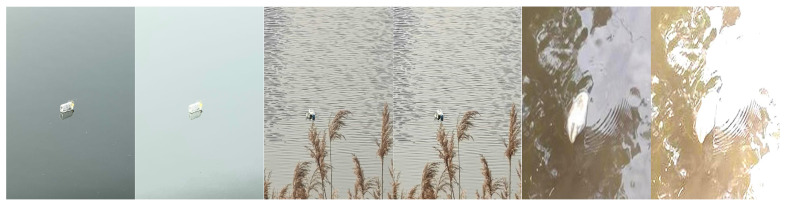
Data augmentation.

In the data preprocessing step, this study employs random brightness and contrast adjustment for data augmentation. Brightness adjustment is performed using PyTorch 2.0.0’s transforms. ColorJitter to randomly adjust the RGB channel values within the range of 0.5 to 1.5, simulating different lighting conditions. To address the common low-contrast issue in water scenes, and to avoid color separation and noise caused by global enhancement methods, a local contrast enhancement algorithm is used for automatic color balancing and adaptive histogram equalization. This helps highlight local details and improve image quality. Simultaneously, optical interference is simulated by synthesizing overexposure and flares, and incorporating highlighted regions at random positions to enhance the model’s robustness to water surface glare and specular reflection. All data augmentation operations are applied after the dataset is partitioned into training, validation, and test sets, and solely to the training set, as shown in Figure 1.

The Space-hehu dataset comprises a total of 28,120 raw images with high-quality annotations. Prior to experimentation, we first randomly divided all raw images into training, validation, and test sets in a ratio of 7:2:1. This resulted in 19,684 training images, 5624 validation images, and 2812 test images. This procedure ensures that the three partitions are independent of each other and is completed before any data augmentation operations, fundamentally preventing information leakage.

**Figure 2:**
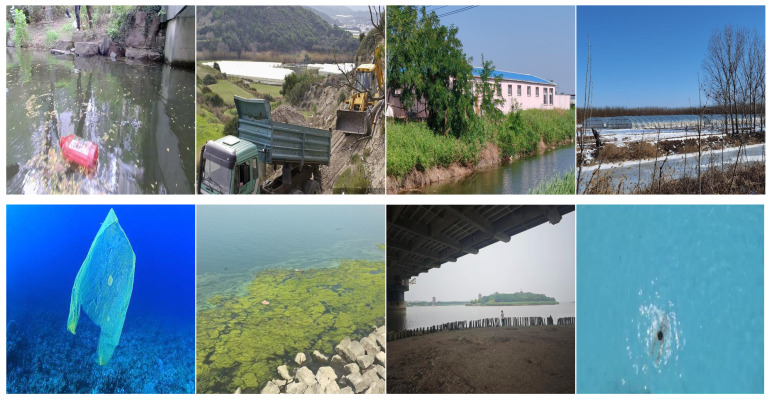
Dataset sample.

**Figure 3:**
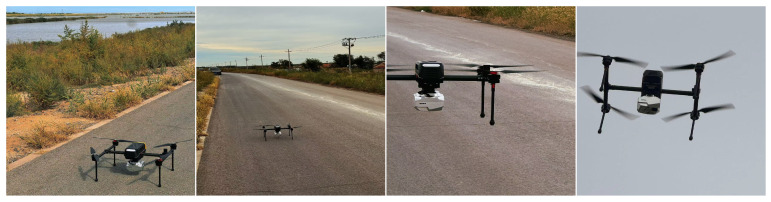
Data Collection Site.

**Figure 4:**
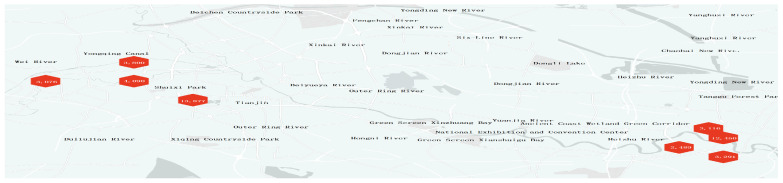
Data Labeling Distribution Map. Red indicates the number of annotations.

During the model training process, the data augmentation strategy is applied only to the images in the training set. Slightly different augmented versions are generated in each training epoch, thereby greatly enhancing the model’s generalization capability. The validation and test sets remain in their original state, undergoing only necessary preprocessing (such as being uniformly resized to 640 × 640 pixels) to objectively evaluate the model’s true performance. Some examples are presented in Figure 2. The data collection site is shown in Figure 3. The number of data annotations is shown in Figure 4. The annotation counts for each category are shown in Table 1.

### 2.3. Parameter Settings

Based on the constructed dataset Space-hehu, this study was conducted. The model training hardware environment was a Lenovo Legion Y9000P computer, equipped with an NVIDIA GeForce RTX 4090 GPU. The operating environment was Windows 11, with an Intel (R) Core (TM) i9-13900HX CPU, an acceleration frequency of 5.8 GHz, a 64-bit processor, and 32 GB of memory. The software environment used the Pytorch-Cuda deep learning framework, Pytorch 2.0.0, with the compiler being Pycharm 2022, and the programming language was Python 3.8.10.

To better and more efficiently identify river and lake spatial targets, the YOLO v11n model was adopted as the main framework, with a batch size of 32 images per batch. The total number of iterations was set to 500 rounds, with the initial learning rate for network weight parameters set at 0.01, and the weight decay rate at 0.0005.

### 2.4. Evaluation Metrics

The evaluation metrics used include Precision (P), Recall (R), mean Average Precision (mAP), Average Precision (AP), F1 score, and Floating Point Operations (FLOPs).

## 3. YOLOv11n-DDHTarget Recognition Model

### 3.1. YOLO v11n Network Architecture

**Figure 5:**
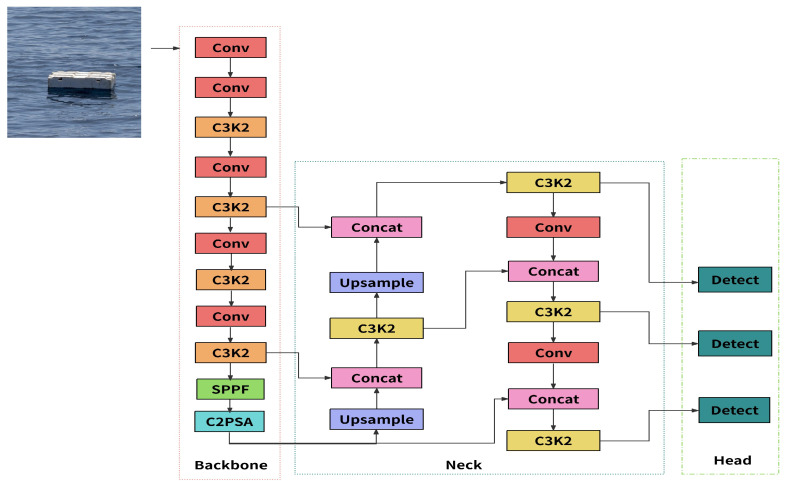
YOLO v11n model structure.

The YOLOv11n model proposed at the YOLO Vision 2024 (YV24) conference [11] represents a significant advancement in real-time object detection. Its network architecture consists of three core modules: Backbone, Neck, and Detect. The Backbone is responsible for feature extraction from images, the Neck is used for multi-scale feature fusion, and the Detect layer performs the object recognition task based on the aforementioned features. The main improvements in YOLOv11n include two aspects: first, the C2 f module in the Backbone and Neck is replaced with C3 K2, optimizing the information flow through feature map segmentation and small convolution kernels, thereby enhancing feature representation efficiency and extraction speed; second, the C2 PSA is introduced after the SPPF module in the Backbone. This module extends from C2 f and integrates the PSA mechanism, improving the model’s ability to perceive critical features. The YOLOv11n network architecture is shown in Figure 5.

The architecture design of YOLOv11n provides an effective technical foundation for monitoring tasks in river and lake water environments. The C3 K2 [12] module in its Backbone enhances multi-scale feature extraction and information flow, improving the ability to recognize targets of different sizes and those in complex backgrounds. This is particularly useful for categories with significant differences, such as scattered construction waste, swimmers, fishing gear, and more. The C2 PSA [13] module introduced after the SPPF employs a pyramid squeeze attention mechanism to strengthen the perception of key areas. It can more accurately capture structural features related to the “Four Chaos” (illegal occupation, illegal mining, random piling, and illegal construction), abnormal water quality regions (such as turbidity and algae aggregation), and the subtle textures of underwater waste. This mechanism effectively suppresses interference in optically complex water environments, improving detection robustness.

These modules work together, enabling YOLOv11n to exhibit excellent perception performance in real-world scenarios such as natural river channels, lakes, and nearshore waters. It can support practical application needs in river and lake monitoring, ecological protection, public safety, and other areas.

### 3.2. Improved YOLOv11n-DDH Network Architecture

**Figure 6:**
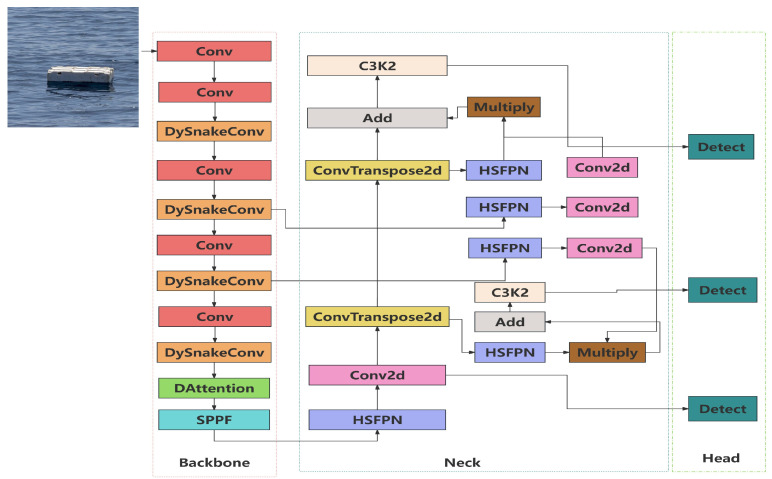
Improved model structure. The arrows in the figure clearly depict the forward propagation of data and the direction of feature flow, while the module color coding visualizes the functional classification.

Compared to other versions of the YOLO series, YOLOv11n performs the best in river and lake target recognition, making it the foundation for further improvements. To address challenges such as varying target scales, diverse shapes, and interference caused by water surface reflections, which lead to difficulties in target recognition and misjudgments in river and lake scenarios, an improved version of YOLOv11n, named YOLOv11n-DDH (model structure shown in Figure 6), is proposed. The YOLOv11n-DDH model introduces dynamic serpentine convolution (DySnakeConv), integrates a deformable attention mechanism (DAttention), and improves the high-level feature selection pyramid network (HSFPN). These enhancements, through edge modeling, dynamic feature selection, and multi-scale noise suppression, collectively improve the model’s ability to distinguish water surface reflections and reduce misjudgment rates. It is suitable for complex scenarios such as waterway target detection and drone aerial photography.

#### 3.2.1. DySnakeConv

DySnakeConv [14] enables convolution kernels to track the trajectory along the target local structure through continuity path constraints and an adaptive coordinate offset mechanism. The core principle is to perform three convolution operations in parallel and concatenate the output features along the channel dimension to integrate multi-directional feature responses. Let the input feature map (x) have the shape C_in_ × H × W, and the kernel size be (k_0_). The three convolution operations are as follows: Standard Convolution (Equation (1)): where (K_0_) is the standard convolution kernel with the shape C_out_ × C_in_ × k × k_0_. Snake Convolution along the x-axis (Equation (2)): where Δx(u, v) is the dynamic offset in the x-direction, deforming the kernel along the horizontal direction. Snake Convolution along the y-axis (Equation (3)): where Δy(u, v) is the dynamic offset in the y-direction, deforming the kernel along the vertical direction. The output feature maps (y_0_), (y_x_), and (y_y_) from the three convolutions are concatenated along the channel dimension (Equation (4)), resulting in a final output (y) with the shape3 C_out_ × H × W.

(1) $$y_{0}^{c_{\mathrm{out}}}\left(i,j\right)=\sum_{c_{m}=1}^{C_{\mathrm{in}}}\sum_{u=-\left|\frac{k}{2}\right|}^{\left\lfloor \frac{k}{2}\right\rfloor }\sum_{v=-\lfloor z\rfloor }^{\left\lfloor \frac{k}{2}\right\rfloor }K_{0}^{c_{\mathrm{out}},c_{\mathrm{in}}}\left(u,v\right)\cdot x^{c_{\mathrm{in}}}\left(i+u,j+v\right)$$

(2) $$y_{x}^{c_{\mathrm{out}}}\left(i,j\right)=\sum_{c_{\mathrm{in}}=1}^{C_{\mathrm{in}}}\sum_{u=-\left\lfloor \frac{k}{2}\right\rfloor }^{\left\lfloor \frac{k}{2}\right\rfloor }\sum_{v=-\left\lfloor \frac{k}{2}\right\rfloor }^{\left\lfloor \frac{k}{2}\right\rfloor }K_{x}^{c_{\mathrm{out}},c_{\mathrm{in}}}\left(u,v\right)\cdot x^{c_{\mathrm{in}}}\left(i+u,j+v+\Delta x\left(u,v\right)\right)$$

(3) $$y_{y}^{c_{\mathrm{ont}}}\left(i,j\right)=\sum_{c_{\mathrm{in}}=1}^{C_{\mathrm{in}}}\sum_{u=-\left|\frac{k}{2}\right|}^{\left\lfloor \frac{k}{2}\right\rfloor }\sum_{v=-\left|\frac{k}{2}\right|}^{\left\lfloor \frac{k}{2}\right\rfloor }K_{y}^{c_{\mathrm{out}},c_{\mathrm{in}}}\left(u,v\right)\cdot x^{c_{\mathrm{in}}}\left(i+u+\Delta y\left(u,v\right),j+v\right)$$

(4) $$y=concat\left(y_{0}^{c_{\mathrm{out}}},y_{x}^{c_{\mathrm{out}}},y_{y}^{c_{\mathrm{out}}},dim=1\right)$$

**Figure 7:**
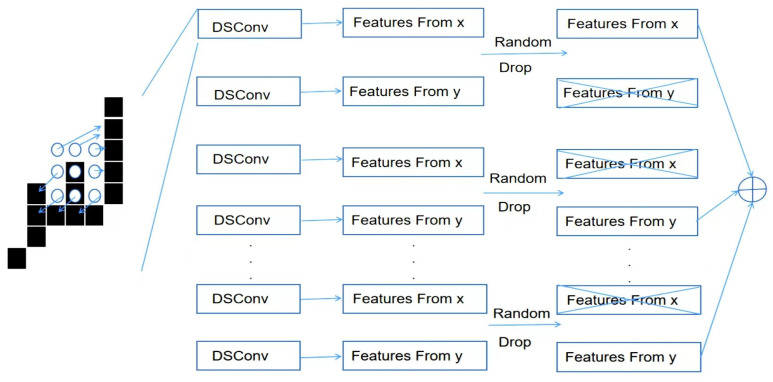
DySnakeConv feature extraction unit. Circles “⊕” represent element-wise addition operations of features.

In a scene with reflections on a lake surface, the edges of real objects and their reflections often exhibit continuous but distorted shapes, such as deformations caused by ripples. By replacing the C3 K2 module of the YOLOv11n base model with the DySnakeConv convolution module, through iterative offset accumulation, forces the convolution kernel to extract features along the edge direction, preventing perceptual domain drift caused by the similarity between reflections and real object textures [15]. In the feature fusion stage, an improved random drop strategy is applied. This strategy randomly removes feature maps from certain views with a certain probability, and then concatenates and accumulates the remaining features. This enhances the model’s robustness, prevents overfitting, and improves generalization ability. The detailed structure is shown in Figure 7.

#### 3.2.2. DAttention

The deformable attention mechanism, DAttention [16], dynamically adjusts the position and range of the attention region by introducing offset sampling points and adaptive weight prediction. This mechanism enables the model to effectively handle complex scenarios such as geometric deformations, varying scales, and partial occlusions, allowing it to focus more precisely on the key semantic areas in an image. DAttention [17] is implemented through the following steps: Input and Grid Generation: Given the input feature map (x ∈ R^H×W×C^), a scaling unit grid (p ∈ R^H^_G_^×W^_G_^×2^) is generated, where (H_G_ = H/r), (W_G_ = W/r) (with (r) as the scaling factor), and the grid coordinates are normalized to the range ([−1, +1]). Query Projection and Offset Calculation: The feature map is linearly projected using a projection matrix (Wq) to obtain the query elements (q = xWq). A lightweight sub-network (θ_offset_) computes the offset (Δp). Key Sampling: The offset is applied to adjust the grid positions to (p + Δp), and bilinear interpolation is used to sample the feature map, yielding the deformed key ($\tilde{k}$ = $\tilde{x}W_{k}$) and value ($\tilde{v}\;=\;\tilde{x}W_{v}$) (Equation (5)), where ($\tilde{x}\;=\;O\left(x,\;p+\Delta p\right)$) (Equation (6)). The interpolation function (O) (Equation (7)) simplifies to a weighted average of the four nearest positions, and the correlation is computed using (g(a, b) = max(0, 1 − ∣a − b∣)). Multi-Head Attention and Position Offsets: Multi-head attention is applied to the query (q), key ($\tilde{k}$), and value ($\tilde{v}$), incorporating relative position offsets ($O(\hat{B};R)$). The output of each attention head is given by Equation (8), where (\sigma) is the activation function, (d) is the dimension parameter, and ($O(\hat{B};R)\;$∈ R^HW×H^_G_^W^_G_) encodes the relative position information. Output Fusion: The outputs of all attention heads are concatenated, and a linear transformation using a weight matrix (Wo) is applied to obtain the final output (z).

(5) $$q=xW_{q},\;\;\tilde{k}=\tilde{x}W_{k},\;\;\tilde{v}=\tilde{x}W_{v}$$

(6) $$with\Delta p=\theta_{offset}\left(q\right),\;\;\tilde{x}=O\left(x,p+\Delta p\right)$$

(7) $$\tilde{x}=O\left(z,\;\left(p_{x},p_{y}\right)\right)=\sum_{\left(r_{x},r_{y}\right)}g\left(p_{x},r_{x}\right)g\left(p_{y},r_{y}\right)z\left[r_{x},r_{z};\right]z^{\left(m\right)}=\sigma \left(\frac{q\;\tilde{k}}{\sqrt{d}}+O\left(\hat{B};R\right)\right){\tilde{v}}^{\left(m\right)}$$

(8) $$z^{\left(m\right)}=\sigma \left|\frac{q^{\left(m\right)}\tilde{k}}{\sqrt{d}}+Q\left(\hat{B};R\right)\right|{\tilde{v}}^{\left(m\right)}$$

**Figure 8:**
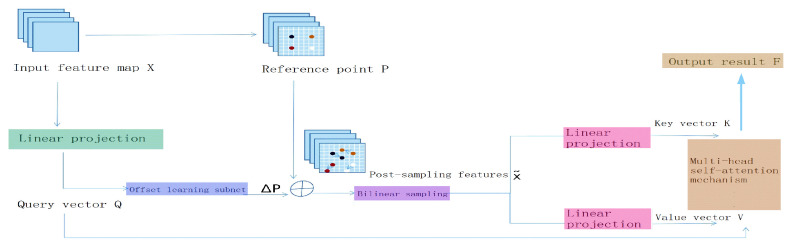
Schematic diagram of the deformable attention mechanism. Circles “⊕” Element-wise addition operation of representative vectors or spatial coordinates.

In the scenario of river and lake environmental monitoring, water surface targets, such as floating objects on the river, often exhibit characteristics like significant scale differences, irregular shapes, and random spatial distribution, which can easily lead to missed detections and misjudgments by recognition models. Replacing the C2 PSA module of the YOLOv11n base model with the DAttention attention mechanism enhances the model’s ability to perceive features of non-rigid targets and suppresses interference from complex backgrounds such as water waves and light reflections, effectively improving the robustness of the model in detecting multi-scale targets. During the feature extraction process, it provides the model with stronger structural perception and context modeling capabilities, significantly improving detection accuracy and generalization performance in complex scenarios. The detailed structure is shown in Figure 8.

#### 3.2.3. HSFPN

**Figure 9:**
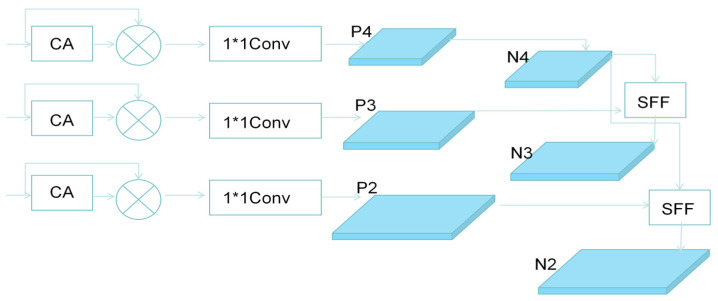
HSFPN network structure. Circles “⊗” Element-wise multiplication (Hadamard product) operation of features.

The High-Level Feature Selection Pyramid Network (HSFPN) [18] improves upon the limitations of traditional feature pyramids by introducing a high-level feature-guided selection mechanism. The core of this mechanism is to use high-level features, which contain rich global semantic information, to filter out low-level features that are rich in details but also noisy, before feature fusion. This process retains only the information beneficial to the object detection task. Specifically, the high-level feature maps, containing image-level contextual semantics, can be used to discriminate potential target distribution areas. HSFPN selectively enhances the low-level features through this high-level semantic information, allowing the fused feature representation to retain both the semantic discriminability of high-level features and the precise localization details from the filtered low-level features [19]. The structure primarily consists of two core sub-modules: feature selection and feature fusion. By selecting and fusing key information from multi-scale features, it generates an optimized feature representation that combines strong semantic representation with high localization accuracy. The detailed structure of HSFPN is shown in Figure 9.

The feature selection module is implemented by placing a 1 × 1 convolution after HSFPN-CA, which flexibly adjusts the number of final output channels to better match the subsequent detection head. It applies a nonlinear transformation to the fused hybrid features, further enhancing their representational capacity [20,21]. This aims to select effective features from multi-scale feature maps and unify their channel dimensions, providing a foundation for subsequent fusion. This module first employs a channel attention mechanism by performing global average pooling and global max pooling on the input features, generating two complementary channel description vectors, which reflect the overall activation level and significant response of the channels, respectively. After merging the two vectors, they are passed through a Sigmoid activation function to generate channel attention weights (*f*_CA_ ∈ R^C×1×1^) [22], enhancing the channels with significant semantic contributions while suppressing noise interference.

**Figure 10:**
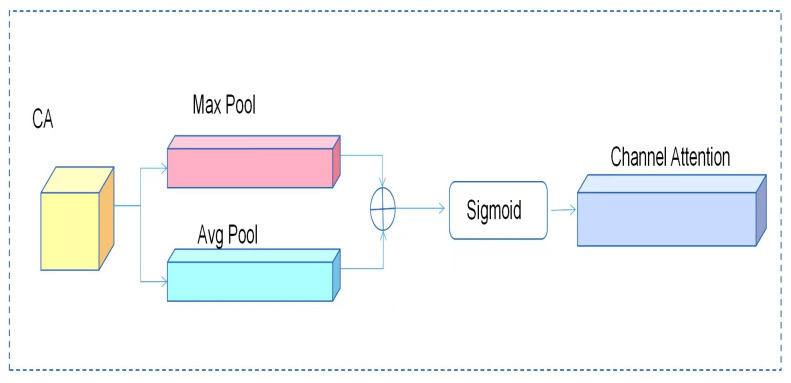
Channel Attention network structure. The circle ‘⊕’ represents the element-wise addition operation of features.

**Figure 11:**
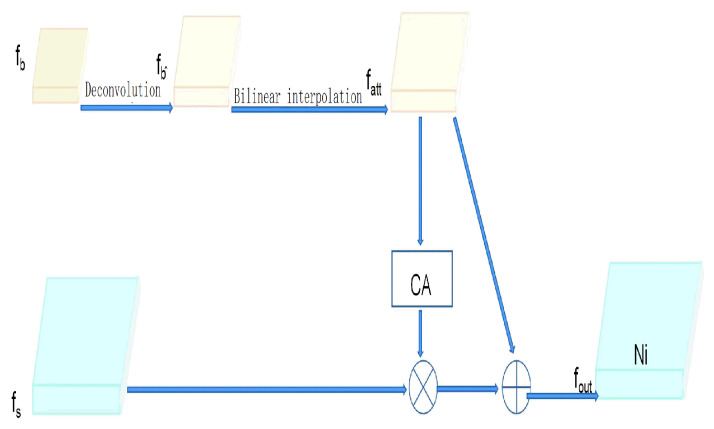
Selet feature fusion module structure. “⊗” denotes element-wise multiplication, used for feature weighting; “⊕” denotes element-wise addition, used for feature integration.

The feature fusion part combines high-level semantic features with low-level spatially detailed features using the Selective Feature Fusion (SFF) [23,24,25] module, which improves localization accuracy while maintaining the high-level semantic expression ability. Specifically, high-level features are upsampled and passed through the channel attention module to generate weights. These weights are applied to the low-level features through pointwise weighting, suppressing semantically irrelevant or noisy responses, and enhancing the target-related regions. Finally, the weighted low-level features are added pointwise to the high-level features to produce the fused feature (*f*_out_) [26]. The detailed structure of the channel attention module is shown in Figure 10, and the detailed structure of the SFF is shown in Figure 11. The feature selection and fusion formulas are as follows (Equations (9) and (10)).

(9) $$f_{att}=BL\left(T-Conv\left(f_{high}\right)\right)f_{out}=f_{low}\times CA\left(f_{att}\right)+f_{att}\;H_{out}=\left(H_{in}-1\right)\times stride\left[0\right]-2\times padding\left[0\right]+ker\;nel\;size\left[0\right]$$

(10) $$W_{out}=\left(W_{in}-1\right)\times stride\left[1\right]-2\times padding\left[1\right]+ker\;nel\;size\left[1\right]$$

## 4. Results and Analysis

### 4.1. Comparison of Different Object Detection Models

Under the same experimental conditions, a comparative analysis of the detection results of various YOLO models based on test set data is shown in Table 2. From Table 2, it can be seen that YOLOv11n outperforms the other baseline models in terms of Recall (R), mAP@50, and F1-score, achieving the highest values. YOLOv10n and YOLOv12n achieve the highest mAP@50-95, but YOLOv11 shows improvements of 0.3 percentage points, 0.2 percentage points, and 2 percentage points over YOLOv10n in R, mAP@50, and F1-score, respectively. YOLOv11n achieves a 1.3 percentage point higher Precision (P) than YOLOv12n. Therefore, YOLOv11n was selected as the baseline model for improvement. The improved model achieves the highest values in Precision (P), Recall (R), mAP@50, mAP@50-95, and F1-score, surpassing the original model by 3.4 percentage points, 2.9 percentage points, 2.5 percentage points, 3.4 percentage points, and 3 percentage points, respectively.

A comparative analysis of detection results among YOLO, RT-DETR, and Faster R-CNN models is conducted. As shown in Table 2, although RT-DETR achieves high accuracy across multiple metrics, demonstrating the powerful capability of the Transformer architecture in global feature interaction and contextual understanding, and its end-to-end nature avoids the hyperparameter tuning and error accumulation associated with NMS, its computational cost is extremely high. The computational load reaches 103.5 GFLOPs, which is more than 17 times that of the other models. This severely limits its deployment in edge device scenarios with constrained computational resources. Compared to YOLOv11n, Faster R-CNN shows lower performance across all metrics. YOLOv11n outperforms it by 4 percentage points in Precision (P), 1.4 percentage points in Recall (R), 2.2 percentage points in mAP@50, 2.9 percentage points in mAP@50-95, and 3 percentage points in the F1 score.

YOLOv11n, while maintaining high precision and average mAP, effectively enhances the recognition and localization accuracy of targets in complex backgrounds, significantly reducing false positives and missed detections. At the same time, the model has a lower computational cost, offering higher operational efficiency under limited hardware resources and placing minimal demands on the processor and memory, thus ensuring the stability of the model. Furthermore, its simple network structure facilitates faster convergence during training, reducing the training difficulty caused by model complexity. Therefore, YOLOv11n was selected as the baseline model for further improvements in this study.

### 4.2. Ablation Experiment

In order to verify that various improvement methods based on the YOLOv11n model can enhance the recognition of river and lake spatial features, 8 sets of ablation experiments were conducted. The first group is the original model, the second, third, and fourth groups added DySnakeConv convolution module, DAttention attention mechanism, and multi-scale feature fusion pyramid HSFPN, respectively, to the first group experiment. The fifth group added the DAttention attention mechanism to the second group experiment, the sixth group added the multi-scale feature fusion pyramid HSFPN to the second group experiment, the seventh group added the multi-scale feature fusion pyramid HSFPN to the third group, and the eighth group is the proposed algorithm in this study, where all three components were incorporated simultaneously. The comparison results are shown in Table 3.

To verify the effectiveness of adding DySnakeConv, the second group of experiments was compared with the first group. The results show that Precision (P), mAP@50, and mAP@50-95 increased by 1.1 percentage points, 0.6 percentage points, and 0.3 percentage points, respectively. This improvement is attributed to the presence of slender, non-rigid targets (such as illegal fishing lines, swimmers’ limbs, and linear features along river boundaries) and weak-texture targets (such as the edges of pollution diffusion) in the Space-hehu dataset. By incorporating DySnakeConv, the model can better capture the detailed features of targets in river and lake scenes, thereby improving detection accuracy.

Compared with the second group of experiments, the fifth group showed improvements of 0.8 percentage points, 2.9 percentage points, 2.1 percentage points, 2.4 percentage points, and 3 percentage points in Precision (P), Recall (R), mAP@50, mAP@50-95, and F1 score, respectively. This improvement is due to the DAttention attention mechanism, which enhances the model’s feature discriminability under occlusion, scale variation, and dynamic background conditions by decoupling the spatial-channel perception mechanism.

Compared with the fourth experiment group, the ninth group showed improvements of 1.5 percentage points in Precision (P), 1.7 percentage points in Recall (R), 1.7 percentage points in mAP@50, 1.9 percentage points in mAP@50-95, and 1 percentage point in the F1 score after incorporating the 1 × 1 convolution. Compared with the second group of experiments, in the sixth group, although mAP@50-95 decreased by 0.2 percentage points, Precision (P), Recall (R), mAP@50, and F1 score improved by 0.7 percentage points, 0.2 percentage points, 0.1 percentage points, and 1 percentage point, respectively. This improvement is due to the challenges posed by high-frequency noise interference from water surface wave reflections, the loss of small target features (such as fishing buoys and swimmer heads) caused by excessive downsampling of deep features, and the separation of entities and their reflections due to geometric-semantic mismatches during cross-level feature fusion (e.g., the target and its reflection being separated into different levels). Adding HSFPN can further enhance the model’s ability to recognize river and lake spatial targets.

Compared with the original model, the improved seventh group of experiments showed an increase in Precision (P) and mAP@50, fully demonstrating the effectiveness of the improvements. The final improved model, compared to the original model, achieved increases of 3.4 percentage points, 2.9 percentage points, 2.5 percentage points, 3.4 percentage points, and 3 percentage points in Precision (P), Recall (R), mAP@50, mAP@50-95, and F1 score, respectively.

Based on Table 4, an analysis is conducted on the effectiveness of the DySnakeConv and DAttention modules on challenging subsets for object detection. Comparing all experimental groups comprehensively, in the small object subset, T4 shows significant improvements over T2 and T3, which use only a single module. This indicates that small object detection relies on the joint enhancement of local fine-grained features and global context. DySnakeConv improves sensitivity to object edges, while DAttention helps highlight weakly signaled objects in complex backgrounds. In the slender object subset, T4 achieves an mAP@50 of 0.911, which is 1.2 percentage points higher than T1. Both T2 and T3 outperform the baseline, and T4 further pushes the performance boundary, demonstrating that the combination of deformable convolution and attention mechanisms can more effectively model the continuous structure and sparse feature distribution of slender objects. In the strong reflection subset, T4 reaches an mAP@50 of 0.878, representing a 2.6 percentage point improvement over T1. Strong reflective surfaces often cause texture distortion and edge blurring. DySnakeConv adapts flexibly to shape distortions under reflection interference through its adaptable receptive field, while DAttention suppresses interference from reflection noise. Their synergy enhances robustness in high-reflection environments.

### 4.3. Comparison of Attention Mechanisms

From Table 5, it can be seen that incorporating the attention mechanism improves P, R, and mAP@50. The core function of the attention mechanism lies in simulating the resource allocation strategy in human cognition, dynamically calculating weights to focus differentially on input information. It computes the relevance scores between the query vector and the key vector, then generates a weighted value vector summary, allowing the model to focus limited computational resources on key parts of the data with higher information density when processing sequences or sets. This mechanism effectively addresses the information bottleneck problem encountered by traditional encoder–decoder structures (e.g., RNNs) when handling long sequences, significantly enhancing the ability to model long-range dependencies and improving model interpretability. In tasks such as machine translation and image caption generation, the attention mechanism not only enhances performance but also provides insights into the model’s decision-making process, marking an important paradigm shift from static representations to dynamic context-awareness.

Among the variants, DAttention performs best in P and mAP@50, with scores of 0.857 and 0.817, respectively. SimAM performs best in R with a score of 0.768. SimAM, CPCA, and MLCA all show an F1 score of 0.79, while P shows a slight decline compared to the original model, whereas DAttention still maintains an F1 score of 0.80. Therefore, DAttention is chosen for improvement, and the modified model achieves the highest values in P and mAP@50, surpassing the original model by 0.7 percentage points and 0.3 percentage points, respectively.

Based on the analysis of Table 6, DySnakeConv achieves the best performance in Precision (P) and mAP@50, with values of 0.861 and 0.820, respectively. This represents an improvement of 1.1 percentage points in P and 0.6 percentage points in mAP@50 compared to YOLOv11n. SCConv achieves a P of 0.852, which is a 0.2 percentage point improvement over YOLOv11n. In contrast, ODConv shows a decline across all metrics: P, R, mAP@50, mAP@50-95, and the F1 score.

Regarding the core evaluation metric mAP@50, DySnakeConv attains the highest performance among all variants, showing a clear improvement over the baseline model. This indicates that it possesses the strongest overall detection capability. In terms of Precision (P), DySnakeConv reaches 0.861, which is significantly higher than the other models. This means it produces the fewest false positives, offering significant practical value for application scenarios where result reliability is paramount. Although its Recall (R) and F1 score show a slight decrease, this is a typical trade-off incurred in the pursuit of higher precision. The improvement in its mAP@50 demonstrates that the gains from increased precision outweigh the loss from a minor drop in recall, resulting in an optimized overall detection performance.

Therefore, considering the need to reduce the false positive rate and improve the confidence of model predictions, the decision was made to select DySnakeConv for enhancement, as it provides the most significant improvement in key precision metrics.

Based on the analysis of Table 7, HSFPN outperforms all other compared structures across key detection metrics. Although AFPN achieves the highest F1 score, and HSFPN’s F1 value is on par with the YOLOv11n model, the F1 score—as the harmonic mean of Precision (P) and Recall (R)—may not increase significantly when both P and R improve and are close in value. While maintaining a comparable F1 score, HSFPN comprehensively improves P, R, and the mAP series, indicating that its enhancement is not merely an optimization of a single metric but rather a systematic improvement in overall detection performance.

HSFPN surpasses BiFPN across all metrics, with particularly notable gains in P and mAP@50, demonstrating that HSFPN holds advantages in feature selection and information retention. Compared to AFPN, HSFPN shows significantly better performance in core metrics such as P, R, and mAP, with only a slightly lower F1 score. However, its mAP@50-95 is 2.2 percentage points higher, proving that HSFPN is more effective in cross-scale feature fusion and semantic consistency processing. The higher mAP@50-95 indicates that HSFPN has stronger adaptability to complex scenes, making it more suitable for multi-scale object detection requirements in practical applications.

### 4.4. Training Results and Analysis

**Figure 12:**
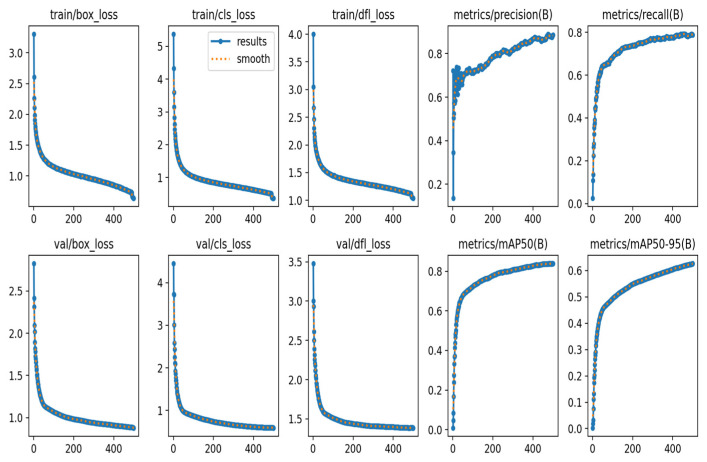
The curve of the model training result.

The loss function curve plays a crucial diagnostic and evaluative role during the model training process. It is not a single curve, but rather a dynamic spectrum composed of multiple sub-loss components, including localization loss, confidence loss, and classification loss. By monitoring its convergence trend, one can visually assess the stability of the training process, convergence efficiency, and the optimization potential of the final model. Specifically, a smooth decline in the total loss curve indicates effective learning of the model parameters. The relative magnitude and convergence speed of each sub-loss component reveal the model’s balance in learning different tasks, such as “localization,” “distinguishing foreground and background,” and “class recognition.” If the classification loss converges too early while the localization loss continues to oscillate, it suggests possible issues with gradient competition or scale imbalance, providing crucial empirical evidence for hyperparameter tuning (such as adjusting loss weight coefficients) and model structure improvements. Figure 12 shows the training and validation loss curves for 500 epochs, which visually reflect the model’s training progress and validation performance, as well as the changes in P, R, mAP@50, and mAP@50-95 before and after the improvements.

### 4.5. Comparison of Detection Accuracy by Category for the Improved Model

Based on the dataset Space-hehu, a comparison of the application effects between the YOLO v11n-DDH model, the baseline model YOLO v11n, and the improved model is shown in Table 8. After adding the DySnakeConv module, the precision of most categories improved, especially for the categories “clean,” “turbid,” “pollute,” and “normal,” which increased by 11.1, 5.9, 4.1, and 3.7 percentage points, respectively. This indicates that DySnakeConv helps in extracting and enhancing image features. Although the improvement for some categories is relatively small, the overall performance shows a noticeable enhancement.

After further introducing the DAttention module, the improvement in precision is not as significant as with the DySnakeConv module. However, for certain specific categories, such as “swimming,” “trash,” and “greenhouse,” precision still increased by 3, 4.6, and 5.7 percentage points, respectively. This indicates that the DAttention module can effectively enhance the model’s discriminative ability for certain complex categories.

High-precision categories, such as “garbage,” “engineering,” “swimming,” and “fishing,” maintain a relatively high precision across all models and demonstrate very stable performance in this study’s algorithm. Notably, for the “engineering” category, the difference between the proposed algorithm and other improved models is minimal, maintaining a high level of 0.989.

Medium-precision categories, such as “bottle,” “trash,” “building,” “plastic,” and “branch,” show some fluctuation in precision across different models. Particularly for the “building” and “branch” categories, their precision improved in the final model, reaching 0.9 and 0.884, respectively. Meanwhile, the detection precision for the underwater target “plastic” has improved across all modified models.

Low-precision categories, such as “clean,” “turbid,” “pollute,” and “normal,” generally exhibit lower precision. Specifically, for the “clean” category, the precision significantly improved with model enhancements, rising from 0.63 in YOLOv11n to 0.826 in the proposed algorithm. This indicates that the proposed algorithm has certain advantages in addressing low-precision categories and demonstrates stronger robustness when handling complex backgrounds.

Overall, by comparing the precision of different models, the proposed algorithm in this study demonstrates significant advantages in most categories, especially in certain specific categories, such as water quality monitoring, where substantial improvements are observed. The improved model in this study exhibits strong generalizability and high precision, making it suitable for a variety of different scenarios and categories, with promising application prospects.

**Figure 13:**
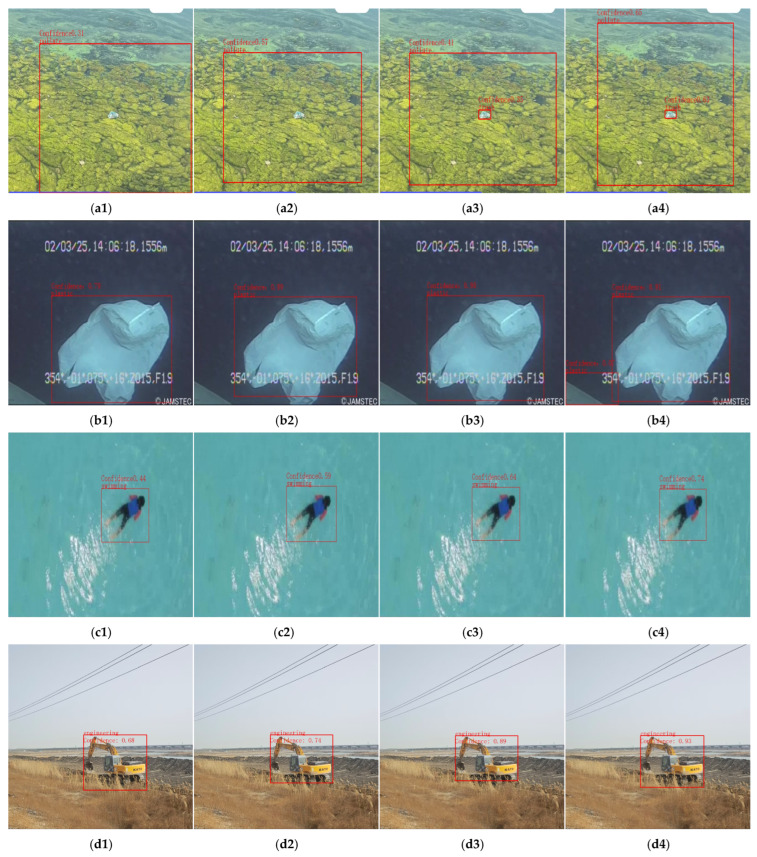

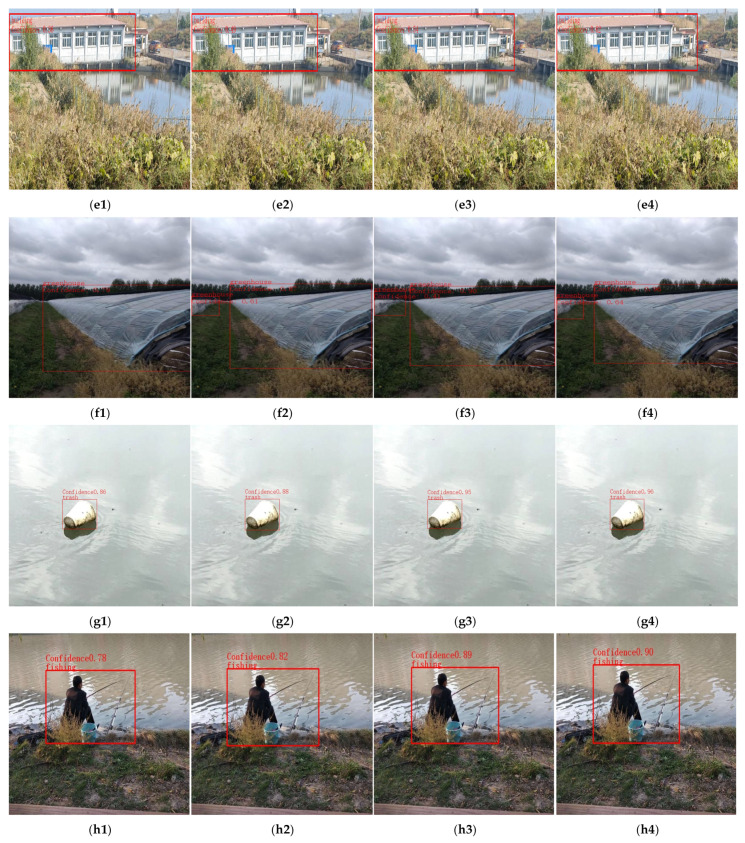

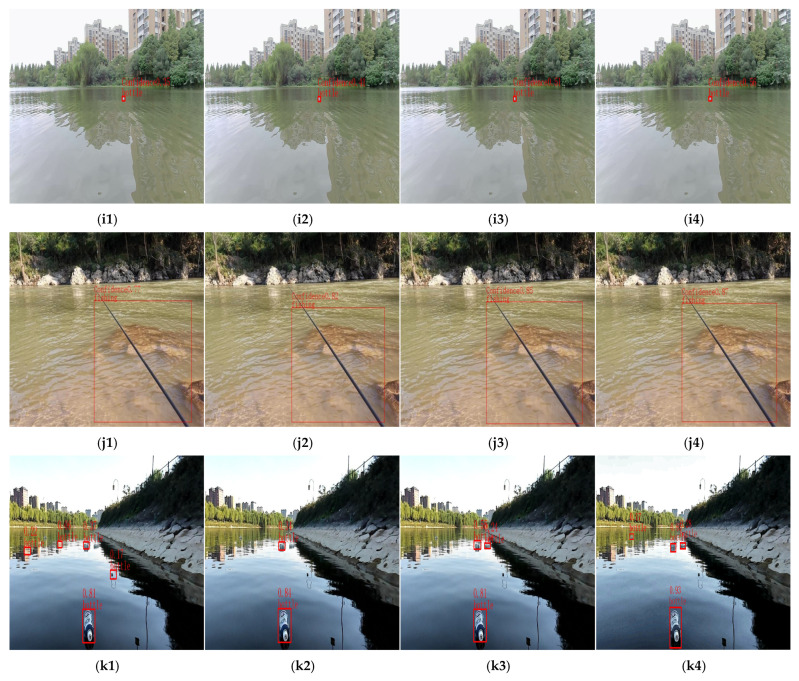
Results of river and lake spatial recognition and detection. (**a1**) YOLO v11n pollute detection results; (**a2**) YOLO v11n + DySnakeConv pollute detection results; (**a3**) YOLOv11n + DySnakeConv + DAttention pollute detection results; (**a4**) YOLO v11n-DDH pollute detection results; (**b1**) YOLO v11n plastic detection results; (**b2**) YOLO v11n + DySnakeConv plastic detection results; (**b3**) YOLOv11n + DySnakeConv + DAttention plastic detection results; (**b4**) YOLO v11n-DDH plastic detection results; (**c1**) YOLO v11n swimming detection results; (**c2**) YOLO v11n + DySnakeConv swimming detection results; (**c3**) YOLOv11n + DySnakeConv + DAttention swimming detection results; (**c4**) YOLO v11n-DDH swimming detection results; (**d1**) YOLO v11n engineering detection results; (**d2**) YOLO v11n + DySnakeConv engineering detection results; (**d3**) YOLOv11n + DySnakeConv + DAttention engineering detection results; (**d4**) YOLO v11n-DDH engineering detection results; (**e1**) YOLO v11n building detection results; (**e2**) YOLO v11n + DySnakeConv building detection results; (**e3**) YOLOv11n + DySnakeConv + DAttention building detection results; (**e4**) YOLO v11n-DDH building detection results; (**f1**) YOLO v11n greenhouse detection results; (**f2**) YOLO v11n + DySnakeConv greenhouse detection results; (**f3**) YOLOv11n + DySnakeConv + DAttention greenhouse detection results; (**f4**) YOLO v11n-DDH greenhouse detection results; (**g1**) YOLO v11n trash detection results; (**g2**) YOLO v11n + DySnakeConv trash detection results; (**g3**) YOLOv11n + DySnakeConv + DAttention trash detection results; (**g4**) YOLO v11n-DDH trash detection results; (**h1**) YOLO v11n fishing detection results; (**h2**) YOLO v11n + DySnakeConv fishing detection results; (**h3**) YOLOv11n + DySnakeConv + DAttention fishing detection results; (**h4**) YOLO v11n-DDH fishing detection results; (**i1**) YOLO v11n bottle detection results; (**i2**) YOLO v11n + DySnakeConv bottle detection results; (**i3**) YOLOv11n + DySnakeConv + DAttention bottle detection results; (**i4**) YOLO v11n-DDH bottle detection results; (**j1**) YOLO v11n fishing detection results; (**j2**) YOLO v11n + DySnakeConv fishing detection results; (**j3**) YOLOv11n + DySnakeConv + DAttention fishing detection results; (**j4**) YOLO v11n-DDH fishing detection results; (**k1**) YOLO v11n bottle detection results; (**k2**) YOLO v11n + DySnakeConv bottle detection results; (**k3**) YOLOv11n + DySnakeConv + DAttention bottle detection results; (**k4**) YOLO v11n-DDH bottle detection results.

This study compares the performance of the YOLO v11n-DDH model, the base YOLO v11n model, and the improved stage models in different object recognition tasks, as shown in Figure 13. Specifically, in water pollutant identification tasks, the YOLO v11n model is only capable of detecting water pollution areas, while it tends to miss floating garbage targets on the water surface. In contrast, the YOLO v11n-DDH model can accurately identify both water quality pollution areas and floating garbage on the water surface. Additionally, the improved model has been enhanced to effectively detect partially occluded structures of greenhouses, effectively addressing the missed detection issues of the base model.

In the identification of floating objects on river surfaces, particularly under strong reflections, the improved model effectively reduces misidentification caused by water surface reflections. In Figure 13(k1), reflections of a riverside house and streetlamp on the water surface were mistakenly identified as floating objects. Figure 13(k2,k3) correctly identified 2 and 3 targets, respectively. The YOLO v11n-DDH model successfully and accurately identified all 4 floating objects on the river surface, enhancing the model’s discernment capability in complex water surface scenarios and significantly reducing false positives from reflections. The recognition accuracy for both extremely small objects and slender objects has also shown a clear improvement.

In terms of underwater plastic identification, as a target recognition task in underwater scenarios, the model exhibits no performance degradation and generally demonstrates consistent improvement. Recognition accuracy for swimmers, houses, and excavators has also improved to varying degrees. Overall, the YOLO v11n-DDH model shows relatively superior recognition accuracy and false positive correction capability across several tasks.

## 5. Limitations

The current system’s image recognition capability mainly relies on conventional RGB image information and has not yet integrated hyperspectral data, resulting in certain functional limitations in water body environmental monitoring. Particularly in water quality analysis and assessment, existing methods can only identify apparent surface pollution in water bodies, making it difficult to achieve fine-grained recognition and quantitative inversion of water quality parameters (such as chlorophyll, suspended solids, colored dissolved organic matter, etc.). As a result, there is a clear lag in the early detection and dynamic perception of pollution events. Additionally, the system currently lacks the capability to trace the sources of pollution, making it unable to effectively analyze the spatial distribution and migration pathways of pollutants, which limits its decision-support value in practical environmental management.

The construction goal of this dataset is to address the problem of multi-target detection and state analysis in real, open-water environments. However, an issue of class imbalance in the annotations persists. Future data collection will prioritize rare and critical scenarios that are difficult to acquire, rather than simply repeating the collection of high-frequency categories.

On the other hand, existing algorithms have not yet achieved deployment on lightweight UAVs and USVs at the edge, making it difficult to meet the requirements for real-time, on-site processing. This limitation constrains the broader application and promotion of the system in field or complex on-site environments.

Furthermore, in terms of domain adaptation, current methods are trained only on selected typical categories and fail to cover the full range of possible class distributions in the target domain. Consequently, the model’s generalization capability remains somewhat limited when encountering unseen categories or data distributions.

## 6. Conclusions

Experiments on the self-built dataset show that the precision, recall, and mAP of the YOLO v11n-DDH model reached 88.4%, 78.9%, and 83.9%, respectively, which is an improvement of 3.4, 2.9, and 2.5 percentage points compared to the original model. Among them, DySnakeConv increased mAP@50 by 0.6 percentage points, DAttention improved mAP@50 by 0.3 percentage points, and HSFPN contributed to a 0.9 percentage point increase in mAP@50.

The improved model effectively suppressed misjudgments caused by water surface reflections, significantly enhancing the accuracy and efficiency of target recognition and positioning within river and lake spatial areas. The river and lake spatial patrol system based on the improved model effectively improves the target recognition efficiency within the monitored water area of rivers and lakes.

## Figures and Tables

**Table 1:** Attributes and annotation counts for different categories.

Classes	Number of Annotations
bottle	2560
packaging	1750
branch	2074
lotus leaf	2332
trash	2778
engineering	3800
greenhouse	4090
building	3076
garbage	2383
fishing	12,450
drowning	951
swimming	2165
clean	589
turbid	587
pollute	746
normal	567
plastic	3291

**Table 2:** Comparison of different models on the test set.

Model	P	R	mAP@50	mAP@50-95	F1	FLOPs	End-to-End Latency
YOLOv3-tiny	0.859	0.737	0.795	0.575	0.79	14.3 GFLOPs	0.9 ms
YOLOv5n	0.825	0.72	0.78	0.55	0.76	5.8 GFLOPs	0.8 ms
YOLOv6n	0.783	0.707	0.756	0.539	0.73	11.5 GFLOPs	1.0 ms
YOLOv8n	0.844	0.752	0.805	0.579	0.79	6.8 GFLOPs	0.9 ms
YOLOv10n	0.827	0.757	0.812	0.599	0.78	8.3 GFLOPs	0.7 ms
YOLOv11n	0.85	0.76	0.814	0.593	0.80	6.3 GFLOPs	0.8 ms
YOLO v11n-DDH	0.884	0.789	0.839	0.627	0.83	5.9 GFLOPs	1.1 ms
YOLOv12n	0.837	0.779	0.823	0.599	0.80	5.8 GFLOPs	2.1 ms
RT-DETR	0.877	0.823	0.854	0.625	0.85	103.5 GFLOPs	3.0 ms
Faster R-CNN	0.81	0.746	0.792	0.564	0.77	5.9 GFLOPs	1.0 ms

**Table 3:** Detection performance of different improvement methods applied to the YOLO v11n model.

ID	Model	DySnakeConv	DAttention	HSFPN	P	R	mAP@50	mAP@50-95	F1	End-to-End Latency
T1	YOLOv11n	×	×	×	0.85	0.76	0.814	0.593	0.80	0.8 ms
T2	YOLOv11n + DySnakeConv	√	×	×	0.861	0.753	0.82	0.596	0.79	1.2 ms
T3	YOLOv11n + DAttention	×	√	×	0.857	0.758	0.817	0.589	0.80	0.9 ms
T4	YOLOv11n + HSFPN + 1*1 Conv	×	×	√	0.856	0.762	0.823	0.599	0.80	0.8 ms
T5	YOLOv11n + DySnakeConv + DAttention	√	√	×	0.869	0.782	0.841	0.62	0.82	1.1 ms
T6	YOLOv11n + DySnakeConv + HSFPN	√	×	√	0.868	0.755	0.821	0.594	0.80	1.0 ms
T7	YOLOv11n + DAttention + HSFPN	×	√	√	0.857	0.764	0.821	0.595	0.80	0.9 ms
T8	YOLO v11n-DDH	√	√	√	0.884	0.789	0.839	0.627	0.83	1.1 ms
T9	YOLOv11n + HSFPN			√	0.841	0.745	0.806	0.58	0.79	1.4 ms

Note: “√” Represents the use of this module.

**Table 4:** Detection performance on difficult subsets.

ID	Model	Small Object Subset—Distant Floating Bottles (mAP@50)	Slender Object Subset—Fishing Lines (mAP@50)	Strong Reflection Subset (mAP@50)	Small Object Subset—Distant Floating (mAP@50-95)	Slender Object Subset—Fishing Lines (mAP@50-95)	Strong Reflection Subset (mAP@50-95)
T1	YOLOv11n	0.82	0.899	0.852	0.513	0.742	0.662
T2	YOLOv11n + DySnakeConv	0.823	0.901	0.86	0.525	0.742	0.674
T3	YOLOv11n + DAttention	0.813	0.9	0.859	0.523	0.734	0.659
T4	YOLOv11n + DySnakeConv + DAttention	0.874	0.911	0.878	0.554	0.754	0.677

**Table 5:** Comparison Results of Attention Mechanism Experiments.

Model	P	R	mAP@50	mAP@50-95	F1	End-to-End latency
YOLOv11n	0.85	0.76	0.814	0.593	0.80	0.8 ms
YOLOv11n + SimAM	0.827	0.768	0.812	0.59	0.79	0.8 ms
YOLOv11n + CPCA	0.837	0.764	0.814	0.588	0.79	0.9 ms
YOLOv11n + DAttention	0.857	0.758	0.817	0.589	0.80	0.9 ms
YOLOv11n + MLCA	0.832	0.762	0.808	0.589	0.79	0.8 ms
YOLOv11n + CAFM	0.835	0.747	0.805	0.576	0.78	0.9 ms

**Table 6:** Results of the Convolution Comparison Experiment.

Model	P	R	mAP@50	mAP@50-95	F1	End-to-End Latency
YOLOv11n	0.85	0.76	0.814	0.593	0.80	0.8 ms
YOLOv11n + DySnakeConv	0.861	0.753	0.82	0.596	0.79	1.2 ms
YOLOv11n + SCConv	0.852	0.76	0.819	0.598	0.80	2.1 ms
YOLOv11n + ODConv	0.838	0.749	0.807	0.575	0.78	1.3 ms

**Table 7:** Results of Multi-Scale Feature Fusion Comparison Experiments.

Model	P	R	mAP@50	mAP@50-95	F1	End-to-End Latency
YOLOv11n	0.85	0.76	0.814	0.593	0.80	0.8 ms
YOLOv11n + HSFPN	0.856	0.762	0.823	0.599	0.80	0.8 ms
YOLOv11n + BIFPN	0.833	0.761	0.811	0.59	0.79	1.0 ms
YOLOv11n + AFPN	0.826	0.743	0.792	0.577	0.81	1.7 ms

**Table 8:** Comparison of results before and after model improvement.

Classes	YOLOv11n	YOLOv11n + DySnakeConv	YOLOv11n + DySnakeConv + DAttention	YOLO v11n-DDH
bottle	0.886	0.898	0.907	0.901
packaging	0.92	0.895	0.922	0.927
branch	0.824	0.862	0.854	0.884
lotus leaf	0.908	0.894	0.906	0.918
trash	0.864	0.833	0.879	0.884
engineering	0.982	0.976	0.99	0.989
greenhouse	0.799	0.8	0.857	0.885
building	0.859	0.869	0.886	0.9
garbage	0.997	0.997	0.995	0.98
fishing	0.929	0.921	0.944	0.944
drowning	0.934	0.899	0.927	0.946
swimming	0.933	0.914	0.944	0.95
clean	0.63	0.741	0.739	0.826
turbid	0.704	0.763	0.754	0.757
pollute	0.735	0.776	0.736	0.728
normal	0.697	0.734	0.677	0.76
plastic	0.855	0.862	0.859	0.857

## Data Availability

The data supporting the findings of this study are available from the authors upon reasonable request. Requests for data should be directed to liuling@tjau.edu.cn with a clear description of the intended use.

## References

[1] Li X., Liu M., Zhang L. (2025). Construction of an Assessment Index System for the “Four Disorder Clearance” Work in Rural Rivers and Lakes. Water Conserv. Technol. Superv..

[2] Wang C. (2025). Assessment of River Ecosystem Health and Methods for Monitoring the Effects of River Management. Value Eng..

[3] Yu S., Yang H., Kong F., Xiong L. (2019). A visual detection method for floating targets on the water surface. Mech. Electr. Eng. Technol..

[4] Tang W., Liu S., Gao H., Tao Q. (2019). Vision-based target detection algorithm for water surface garbage cleaning robots. Sci. Technol. Eng..

[5] Li F. (2017). Research on the Optimization of Water Quality Monitoring Algorithm and System Integration in Dynamic Scenes.

[6] Redmon J., Divvala S., Girshick R., Farhadi A. (2016). You only look once: Unified, real-timeobject detection. IEEE Conference on Computer Vision Andpattern Recognition.

[7] Song P., Li P., Dai L., Wang T., Chen Z. (2023). Boosting R-CNN: Reweighting R-CNN samples by RPN’s error forunderwater object detection. Neurocomputing.

[8] Xu J., Chen C., Zhang S. (2024). Research on small target detection of waterborne garbage based on lightweight algorithms. Electron. Meas. Technol..

[9] Wang Z., Peng T. (2025). Improved YOLOv8n Model Method for Grape Leaf Disease Identification. J. Yibin Univ..

[10] Wang Y., Li X. (2025). Research on Gesture Recognition Based on Improved YOLOv5s Algorithm. J. Luoyang Inst. Sci. Technol. Nat. Sci. Ed..

[11] Jocher G., Qiu I. Ultralytics YOLO11[EB/OL]. https://github.com/ultralytics/ultralytics.

[12] Khanam R., Hussain M. (2024). YOLOv11: An overview of the key architectural enhancements[EB/OL]. arXiv.

[13] Sharma A., Kumar V., Longchamps L. (2024). Comparative performance of YOLOv8, YOLOv9, YOLOv10, YOLOv11 and Faster R-CNN models for detection of multiple weed species. Smart Agric. Technol..

[14] Yu W., Xie B., Liu D., Fang C., Zhang J. (2024). Casdenet:Cascade Automatic Road Detection Network Based on Dynamic Snake Convolution and Edge Branch. IGARSS 2024—2024 IEEE International Geoscience and Remote Sensing Symposium.

[15] Zhang W. (2025). Improved UNet3 Road Extraction Method for Remote Sensing Images Based on Serpentine Dynamic Convolution. Surv. Spat. Geogr. Inf..

[16] Xia Z., Pan X., Song S., Li L.E., Huang G. (2022). Vision Transformer with Deformable Attention. 2022 IEEE/CVF Conference on Computer Vision and Pattern Recognition.

[17] Shi Y., Zheng S., Bian M., Zhang X., Yang L. (2025). DASS-YOLO: Improved YOLOv7-Tiny with Attention-Guided Shape Awareness and DySnakeConv for Spray Code Defect Detection. Symmetry.

[18] Chen Y., Zhang C., Chen B., Huang Y., Sun Y., Wang C., Fu X., Dai Y., Qin F., Peng Y. (2024). Accurate leukocyte detection based on deformable-DETR and multi-level feature fusion for aiding diagnosis of blood diseases. Comput. Biol. Med..

[19] Chen L., Wu L., Ren Q. (2025). A multimodal data fusion-based intelligent detection method for lump coal on underground conveyor belts in smart manufacturing. J. Ind. Inf. Integr..

[20] Zhang X. (2024). Research on Marine Organism Detection Technology Based on Deep Learning. Master’s Thesis.

[21] Liu W. (2025). Surface Defect Detection of Steel Based on Deep Learning.

[22] Hu J., Shen L., Sun G. Squeeze-and-Excitation Networks. Proceedings of the 2018 IEEE/CVF Conference on Computer Vision and Pattern Recognition.

[23] Tan M., Pang R., Le Q.V. Efficientdet: Scalable and efficient objectdetection. Proceedings of the 2020 IEEE/CVF Conference on Computer Vision and Pattern Recognition.

[24] Islam M.P., Hatou K., Shinagawa K., Kondo S., Kadoya Y., Aono M., Kawara T., Matsuoka K. (2026). Hort-YOLO: A multi-crop deep learning model with an integrated semi-automated annotation framework. Comput. Electron. Agric..

[25] Ren Z. (2025). Research on Student Exam Posture Detection Algorithm Based on Improved YOLOv8. Master’s Thesis.

[26] Guo X. (2025). Tea Bud Detection Method Based on Deep Learning. Master’s Thesis.

